# Influence of Ecological Zones and Honey Bee Morphometric Traits on the Physicochemical Properties of Honey in Kazakhstan

**DOI:** 10.3390/foods15142454

**Published:** 2026-07-10

**Authors:** Maxat Toishimanov, Ulzhan Nuraliyeva, Gaukhar Moldakhmetova, Merey Torekhanov, Zhanar Sheralieva, Gulim Khalykova, Nuradil Spatay, Anton Skryl, Timur Krupskiy, Kanat Mustafin

**Affiliations:** 1 Food and Environment Safety Laboratory, Kazakh National Agrarian Research University, Almaty 050010, Kazakhstan; 2 Kazakh Research Institute of Livestock and Fodder Production, Almaty 050035, Kazakhstan; 3 Department of Zooengineering and Biotechnology, Kazakh National Agrarian Research University, Almaty 050010, Kazakhstan

**Keywords:** honey quality, *Apis mellifera*, physicochemical properties, ecological zones, honey authentication, honey bee morphometry, geometric morphometrics, Kazakhstan

## Abstract

This study aimed to evaluate the influence of ecological zones and honey bee morphometric characteristics on the physicochemical properties of honey produced in different regions of Kazakhstan. A total of 103 honey samples were collected and analyzed for key quality parameters, including moisture content, sugars, acidity, pH, and diastase activity. Multivariate statistical approaches, including principal component analysis (PCA), linear discriminant analysis (LDA), and multivariate analysis of variance (MANOVA), were applied to assess variability and underlying patterns. Geometric morphometric analysis of wing shape (MorphoJ) revealed a high degree of morphological homogeneity across all samples, with no distinct clustering in PCA space. This was further supported by canonical variate analysis (CVA) using IdentiFly, which assigned the majority of samples to the C lineage (*Apis mellifera carnica*) with high classification probability, indicating a uniform population structure. MANOVA results demonstrated that neither ecological zone nor morphometric traits exerted a significant global effect on honey physicochemical properties (*p* > 0.05). However, significant interaction effects were identified between ecological zone and specific morphometric variables, particularly sternite length and cubital index (*p* < 0.05), suggesting a context-dependent influence.

## 1. Introduction

Honey is a natural product with high nutritional and functional value, widely consumed worldwide due to its unique composition and bioactive properties. It is composed primarily of sugars, organic acids, enzymes, minerals, and phenolic compounds, the proportions of which determine its physicochemical characteristics and quality [1,2,3]. The composition of honey is influenced by multiple factors, including botanical origin, environmental conditions, processing, and storage, making it a complex and variable natural product [4].

The quality and authenticity of honey are commonly evaluated using standardized physicochemical parameters, such as moisture content, reducing sugars, sucrose, pH, free acidity, hydroxymethylfurfural (HMF), and diastase activity, as defined by the Codex Alimentarius and the International Honey Commission (IHC) methods [1,2]. These parameters are widely used not only to assess freshness and quality but also to detect adulteration and ensure compliance with international standards [2]. Numerous studies have demonstrated that these indicators are highly sensitive to environmental and geographical conditions, including climate, altitude, and floral diversity [4,5,6].

Environmental factors, particularly ecological zone and botanical origin, play a key role in shaping honey composition. Variations in altitude, temperature, and vegetation type significantly affect nectar composition and, consequently, honey quality [5,6]. For instance, honey produced in mountainous regions often exhibits higher enzymatic activity and acidity, whereas honey from arid and semi-desert regions tends to have lower moisture content and higher pH values [6,7]. In addition, floral diversity influences the concentration of phenolic compounds and antioxidant activity, further contributing to regional variability [8,9]. Although botanical origin is recognized as one of the principal factors influencing honey composition, ecological zones integrate multiple environmental variables, including vegetation type, climate, altitude, and land use, which together shape nectar availability and honey characteristics. Therefore, ecological zoning provides a useful framework for investigating regional variation in honey quality under commercial beekeeping conditions.

In parallel with environmental influences, the biological characteristics of honey bees may also contribute to variability in honey composition. Morphometric analysis of *Apis mellifera* provides valuable information about subspecies differentiation, ecological adaptation, and foraging behavior [10,11,12]. Classical morphometric parameters, such as proboscis length, wing dimensions, and body segment measurements, as well as derived indices including the cubital index and discoidal shift, are widely used to characterize honey bee populations and assess their adaptation to local conditions [10]. Although honey bee morphometric traits are not expected to directly determine honey physicochemical composition, they may reflect ecological adaptation and foraging capacity under different environmental conditions. Morphological characteristics such as wing dimensions, proboscis length, and body size can influence flight performance, foraging range, floral resource accessibility, and nectar collection efficiency. Consequently, these traits may be indirectly associated with regional variation in honey composition through their interaction with ecological conditions and available floral resources. The present study therefore evaluates honey bee morphometric characteristics as ecological indicators rather than direct determinants of honey quality [11,12].

The role of honey bee morphology is particularly relevant in regions with high ecological heterogeneity, such as Central Asia. Kazakhstan represents a unique geographical area characterized by diverse ecological zones, including mountain, foothill, steppe, and semi-desert environments. Recent studies have reported significant variability in both honey composition and honey bee populations across these zones [13,14,15]. In particular, honey samples from Kazakhstan have been successfully classified based on physicochemical and biochemical properties using multivariate approaches, confirming the strong influence of geographical origin [13,14]. At the same time, morphometric and genetic studies indicate the presence of diverse honey bee populations, including the endemic subspecies *Apis mellifera pomonella* and populations with introgression from other lineages [15,16,17,18,19]. This complexity provides an opportunity to investigate the combined effects of environmental and biological factors on honey quality.

Multivariate statistical methods, such as principal component analysis (PCA) and linear discriminant analysis (LDA), are increasingly used to explore the relationships between honey composition, geographical origin, and biological variables [20,21]. These approaches allow for the identification of hidden patterns, classification of samples, and evaluation of the relative contribution of multiple factors to honey variability.

Therefore, the aim of the present study was to evaluate the influence of ecological zones, and honey bee morphometric traits were evaluated together with ecological zone characteristics to explore their associations with honey physicochemical properties. We hypothesized that ecological zones represent the primary source of variation in honey physicochemical properties, whereas honey bee morphometric traits may serve as complementary indicators of ecological adaptation that are associated with, rather than directly responsible for, regional differences in honey composition. The study integrates physicochemical analysis of honey with morphometric characterization of honey bees and applies advanced statistical methods to assess their combined effects. The findings contribute to a better understanding of the interaction between environmental conditions, bee morphology, and honey quality and provide a scientific basis for the characterization and authentication of regional honey.

## 2. Materials and Methods

### 2.1. Sample Collection

A total of 103 honey samples were collected from apiaries located in four ecological zones of Kazakhstan: mountain (*n* = 25), foothill (*n* = 29), steppe (*n* = 27), and semi-desert (*n* = 22) regions. Sampling was conducted during August, corresponding to the peak honey harvesting period. All samples were obtained from managed colonies of *Apis mellifera* maintained under standard beekeeping conditions. Honey samples and honey bee specimens were collected from the same managed colonies at each apiary. For every honey sample, worker bees used for morphometric analysis originated from the corresponding colony from which the honey was harvested, thereby ensuring direct correspondence between the physicochemical and morphometric datasets. Detailed apiary locations are provided in Table S1 (Supplementary Materials). The ecological zones were classified according to the landscape and vegetation typology of Kazakhstan [5]. Honey samples (approximately 500 g each) were collected directly from sealed honeycomb frames using food-grade stainless steel extractors and stored in sealed glass containers at 18–20 °C in the dark until analysis. Immediately after extraction, honey samples were transferred into sterile airtight glass containers, protected from direct sunlight, and transported to the laboratory under ambient conditions on the day of collection. Upon arrival, all samples were stored in the dark at 18–20 °C until analysis. Physicochemical analyses were initiated within two weeks after completion of sample collection. All 103 honey samples were handled, transported, stored, and analyzed under identical conditions to minimize variability associated with storage or thermal history. Apiaries were selected to represent the major ecological zones of Kazakhstan. The surrounding vegetation consisted predominantly of naturally occurring regional flora typical for each ecological zone, including mountain meadows and forest vegetation in mountain areas, mixed herbaceous communities in foothill regions, grassland vegetation in steppe areas, and drought-tolerant xerophytic vegetation in semi-desert regions. Honey samples were collected from commercial apiaries under routine beekeeping management.

### 2.2. Ecological Characterization of Sampling Sites

Sampling sites were selected to represent the major ecological zones of Kazakhstan, encompassing mountain, foothill, steppe, and semi-desert landscapes. These ecological zones differ markedly in altitude, climatic conditions, vegetation structure, and nectar resource availability, providing a broad environmental gradient for evaluating honey quality.

Mountain apiaries (approximately 1200–2200 m above sea level) were located in the Northern Tien Shan and Dzungarian Alatau regions. These areas are characterized by relatively low summer temperatures, higher precipitation, and diverse mountain meadow and forest vegetation. The predominant flowering communities include species belonging to the *Rosaceae*, *Apiaceae*, *Lamiaceae*, *Fabaceae*, and *Asteraceae* families, which provide abundant nectar resources during the main honey flow.

Foothill apiaries (approximately 600–1200 m above sea level) were situated within transitional landscapes between mountain and steppe ecosystems. These regions are characterized by moderate climatic conditions and heterogeneous vegetation consisting of natural grasslands, shrub communities, wild fruit trees, and agricultural crops. The prolonged flowering period and relatively high plant diversity support continuous nectar availability throughout the beekeeping season.

Steppe apiaries (approximately 250–700 m above sea level) were established in open grassland ecosystems dominated by herbaceous vegetation adapted to continental climatic conditions. Typical nectar-producing plants include representatives of the *Asteraceae*, *Fabaceae*, *Brassicaceae*, and *Poaceae*-associated flowering communities. These areas experience warm summers, moderate precipitation, and extensive natural grazing lands.

Semi-desert apiaries (approximately 150–450 m above sea level) were located in arid regions characterized by low annual precipitation, high summer temperatures, and xerophytic vegetation. Dominant nectar-producing flora include *Artemisia* spp., *Tamarix* spp., *Chenopodiaceae*, and other drought-tolerant plant species typical of semi-arid ecosystems. Although floral diversity is generally lower than in mountain and foothill regions, these communities provide important nectar resources during seasonal flowering periods.

To minimize seasonal variability, all honey samples were collected during the principal honey harvesting period in August under routine commercial beekeeping management. Geographic coordinates and approximate altitude of each apiary are provided in Supplementary Table S1. The ecological classification was based on the landscape and vegetation zonation of Kazakhstan and was used to characterize the environmental conditions surrounding each apiary rather than to determine the botanical origin of individual honey samples. Since melissopalynological analysis was beyond the scope of the present study, the vegetation descriptions represent the dominant regional flora expected within each ecological zone and should not be interpreted as evidence of the botanical origin of individual honey samples.

### 2.3. Physicochemical Analysis

Physicochemical analysis was performed according to the harmonized methods of the International Honey Commission (IHC) and the Codex Alimentarius standard for honey [22]. Moisture content was determined by refractometry using a calibrated hand refractometer (Atago, Tokyo, Japan), and results were expressed as percentage (%). Reducing sugars and sucrose content were determined by the Luff–Schoorl method. Free acidity was determined by potentiometric titration and expressed in milliequivalents per kilogram (meq/kg). pH was measured using a calibrated digital pH meter (Mettler-Toledo, Columbus, OH, USA). Diastase activity was determined spectrophotometrically according to the Phadebas method and expressed as the diastase number (DN, Schade units) [22].

### 2.4. Morphometric Analysis

Morphometric analysis was conducted on worker bees collected from the same colonies from which the honey samples were obtained. At each apiary, twenty adult worker bees were randomly collected from each colony during honey sampling and preserved in 70% ethanol until analysis. The number of twenty workers per colony was selected in accordance with established morphometric protocols for honey bee population characterization, providing representative colony-level averages while minimizing within-colony variation. Individual measurements were recorded for each worker, and the mean values were subsequently used for statistical analyses, including the calculation of morphometric indices such as the cubital index, hantel index, and discoidal shift. Measurements were performed under a stereomicroscope (Nikon SMZ745, Tokyo, Japan) equipped with a calibrated ocular micrometer. The following standard morphometric parameters were recorded following the protocol of Ruttner [23] and Tofilski [20], including proboscis length, tergite and sternite dimensions, wax mirror length and width, tarsus length and width, wing length and width, hamuli count, and tarsal index.

The cubital index (CI) was calculated as the ratio of vein segment a to vein segment b on the anterior wing; the hantel index was computed as the ratio of the widths of the first and second hamuli; and the discoidal shift (DS) was expressed as a positive or negative value depending on the displacement of the discoidal cell [8,23].

Geometric morphometric analysis of honey bee wing shape was performed using MorphoJ version 1.07a (Klingenberg, Manchester, UK). Digital images of the forewings were obtained under standardized conditions, and homologous landmarks were digitized according to established protocols for honey bee wing venation analysis. A Procrustes superimposition was applied to remove non-shape variation (size, orientation, and position), and principal component analysis (PCA) was conducted to evaluate patterns of shape variation among samples [24].

Morphometric classification of honey bee samples was additionally performed using IdentiFly. This software is based on classical wing venation measurements and reference datasets for major evolutionary lineages (A, C, M, and O). Canonical variate analysis (CVA) was applied to assign each sample to a reference group, and classification probabilities were calculated based on Mahalanobis distances [25].

### 2.5. Statistical Analysis

Descriptive statistics (mean ± standard deviation) were calculated for all physicochemical and morphometric variables. Differences among ecological zones were assessed by one-way analysis of variance (ANOVA) with post hoc Tukey’s HSD test at a significance level of *p* < 0.05. Multivariate analysis of variance (MANOVA) was performed using Wilks’ Lambda to evaluate the combined effects of ecological zone, morphometric traits, and morphometric indices on honey physicochemical properties. The objective of the multivariate analyses was to evaluate statistical associations between ecological zones, honey bee morphometric characteristics, and honey physicochemical properties rather than to establish direct causal relationships. Prior to MANOVA, the assumptions of multivariate normality and homogeneity of covariance matrices (Box’s M test) were evaluated. Because these assumptions were not fully satisfied, the MANOVA results were interpreted as an exploratory multivariate analysis. Principal component analysis (PCA) was applied separately to three datasets: (i) honey physicochemical properties, (ii) raw morphometric parameters, and (iii) morphometric indices. Linear Discriminant Analysis (LDA) was performed to evaluate the ability of the selected physicochemical and morphometric variables to discriminate among ecological zones. Model performance was assessed using leave-one-out cross-validation (LOOCV). The predictive robustness of the model was evaluated by reporting the cross-validated confusion matrix together with the overall cross-validated classification accuracy (%), which are presented in the Results and Supplementary Table S2. All statistical analyses were performed in JMP Pro 17 [21,26,27].

## 3. Results

This section presents the results of physicochemical analysis of honey samples, morphometric characterization of honey bees, and multivariate statistical analysis across four ecological zones of Kazakhstan.

### 3.1. Physicochemical Properties of Honey Across Ecological Zones

The physicochemical parameters of honey samples varied significantly across ecological zones (Table 1). Moisture content ranged from 15.8 ± 1.2% (mountain) to 17.2 ± 2.0% (steppe), with steppe honey showing significantly higher moisture than samples from other zones (*p* < 0.05). All values were within the Codex Alimentarius limit of 20% [17]. Reducing sugar content was the highest in foothill honey (82.7 ± 8.9%) and the lowest in semi-desert honey (72.5 ± 5.8%), with significant differences among zones (*p* < 0.05). Sucrose content did not differ significantly among ecological zones (*p* > 0.05), ranging from 2.4 ± 0.6% (mountain) to 3.8 ± 1.5% (steppe), and all values remained below the 5% limit recommended by Codex [17]. Free acidity was the highest in mountain honey (30.5 ± 5.2 meq/kg) and the lowest in semi-desert honey (22.1 ± 6.3 meq/kg), consistent with the typically more acidic character of honey from alpine floral sources [3]. pH values were significantly lower in mountain and foothill zones compared to steppe and semi-desert zones (*p* < 0.05). Diastase activity was significantly higher in mountain (18.4 ± 6.3 DN) and foothill (16.5 ± 7.8 DN) honeys compared to steppe (11.2 ± 5.4 DN) and semi-desert (10.8 ± 4.2 DN) samples (*p* < 0.05), indicating greater enzymatic activity in cooler, higher-altitude environments.

### 3.2. Morphometric Characterization of Honey Bees

Morphometric parameters measured across the four ecological zones are summarized in Table 2. Significant differences among zones were detected for most body measurements (*p* < 0.05). Mountain bees exhibited the largest body dimensions, including the greatest proboscis length (6.69 ± 0.58 mm), tergite length (2.49 ± 0.25 mm), sternite length (3.18 ± 0.32 mm), and wing length (9.77 ± 0.27 mm). Semi-desert bees showed similarly large body size, particularly in sternite dimensions (3.21 ± 0.30 mm) and wing length (9.71 ± 0.32 mm), while steppe bees were consistently smaller across most parameters. Foothill bees occupied an intermediate position. Hamuli count and tarsal index did not differ significantly among zones (*p* > 0.05), suggesting that these traits are less sensitive to environmental variation.

### 3.3. Morphometric Identification

**Figure 1:**
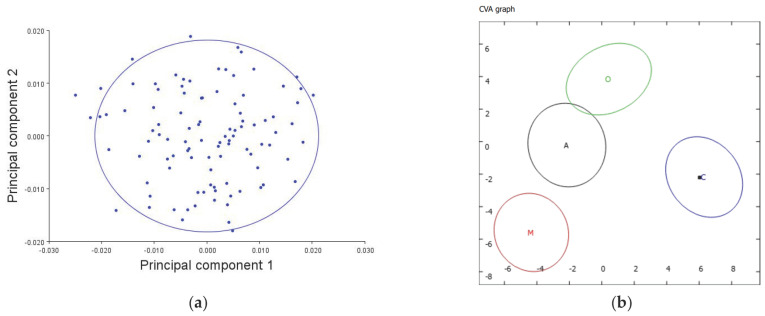
(**a**) Multivariate morphometric analysis of honey bee samples (*n* = 103) based on wing characteristics, combining principal component analysis (PCA, MorphoJ) and (**b**) canonical variate analysis (CVA, IdentiFly). The compact distribution of samples and their consistent classification within the C lineage (*Apis mellifera carnica*) indicate a high degree of morphometric and taxonomic uniformity.

Principal component analysis (PCA) of wing shape variation was performed using MorphoJ software on 103 honey bee samples. The distribution of individuals in the morphospace defined by the first two principal components demonstrated a compact and continuous pattern without distinct clustering (Figure 1a).

The majority of samples were concentrated around the centroid, with only moderate dispersion along both principal axes, indicating limited morphological variability within the studied population. No clear separation of samples into discrete groups was observed, suggesting the absence of morphologically distinct subpopulations.

Morphometric identification of honey bee samples was performed using the IdentiFly software based on wing venation characteristics and canonical variate analysis (CVA). The classification results revealed a clear assignment of the analyzed samples to class C, corresponding to the *Apis mellifera carnica* lineage, with a high probability of 0.824 (Figure 1b).

The CVA score plot further supported this result, showing a distinct separation of the analyzed sample within the cluster corresponding to the C lineage, with no overlap with other subspecies groups. The canonical variate scores (CV1 = 6.03, CV2 = −2.21, CV3 = −0.91) positioned the sample firmly within the C cluster, reflecting strong morphometric conformity with *A. m. carnica*.

Morphometric indices (Table 3) also differed significantly among ecological zones. The cubital index (CI) increased progressively from mountain (2.66 ± 0.04) to semi-desert (2.90 ± 0.06) zones (*p* < 0.05), indicating a gradient in wing venation proportions linked to altitude and climate. The hantel index did not differ significantly among zones (*p* > 0.05), with values ranging from 1.06 to 1.09, suggesting conservative morphological proportions across populations. The discoidal shift was the highest in the semi-desert zone (4.23 ± 0.18) and the lowest in the steppe zone (3.71 ± 0.14), with significant differences among all zones (*p* < 0.05).

### 3.4. Integrated Analysis of Honey Physicochemical Properties and Honey Bee Morphometric Traits

Multivariate analysis of variance (MANOVA) was performed to evaluate the combined effects of ecological zone, morphometric traits, and morphometric indices on honey physicochemical properties.

The overall model, including all predictors, was not statistically significant based on Wilks’ Lambda (Λ = 0.16, F = 1.11, *p* = 0.214), indicating the absence of a global multivariate effect across all variables. Similarly, the main effect of ecological zone was not significant (Λ = 0.853, F = 0.66, *p* = 0.844), suggesting that environmental conditions alone do not explain the variability in honey composition, as shown in Table 4.

Among morphometric traits, most interaction terms were not significant (*p* > 0.05). However, significant multivariate effects were observed for the interaction between ecological zone and proboscis length (Λ = 0.782, F = 1.05, *p* = 0.0411) as well as sternite length (Λ = 0.657, F = 1.84, *p* = 0.0227). These findings indicate that specific body-size-related traits influence honey physicochemical properties in a context-dependent manner.

In contrast, interactions involving tergite dimensions and wing size were not statistically significant, suggesting a limited contribution of these traits to honey variability.

Regarding morphometric indices, a significant interaction effect was identified between ecological zone and cubital index (Λ = 0.008, F = 1.51, *p* = 0.0317), whereas hantel index and discoidal shift showed no significant effects (*p* > 0.05).

**Figure 2:**
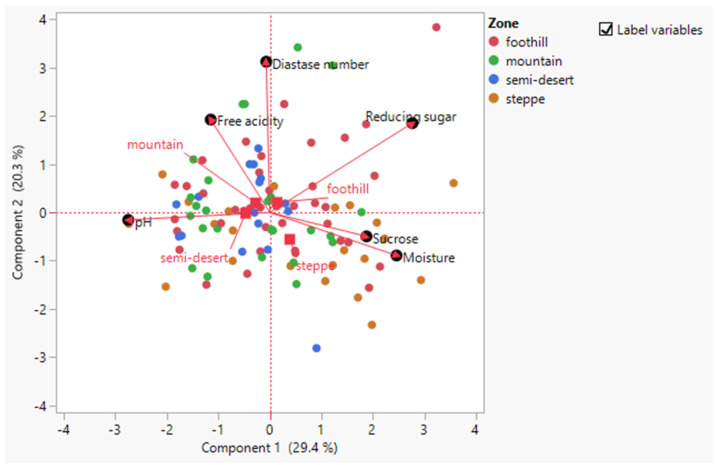
Principal component analysis (PCA) biplot of physicochemical properties of honey samples across ecological zones.

Principal component analysis of the physicochemical characteristics showed that the first principal component (PC1) explained 29.44% of the total variance, whereas the second principal component (PC2) explained 20.31%, together accounting for 49.75% of the overall variability. The first three principal components collectively explained 65.20% of the total variance, indicating that these components captured the major multivariate structure of the dataset (Figure 2).

Inspection of the loading matrix revealed that reducing sugar (0.720) and moisture (0.640) contributed most strongly and positively to PC1, whereas pH (−0.715) showed the strongest negative loading. Therefore, PC1 primarily represented a gradient associated with sugar concentration and moisture content opposed to pH. Along PC2, the largest positive loadings were observed for diastase number (0.814), free acidity (0.501), and reducing sugar (0.482), indicating that enzymatic activity and acidity were the principal factors contributing to sample separation along this axis.

Projection of ecological zones onto the PCA space demonstrated that steppe samples were associated with higher PC1 scores and lower PC2 scores, reflecting their relationship with higher reducing sugar content, whereas mountain samples were positioned toward positive PC2 values, indicating greater association with higher diastase activity and free acidity. Semi-desert samples were primarily located along the negative direction of PC1, corresponding to relatively higher pH values and lower sugar-related loadings.

Inspection of the loading plot indicated that the separation of ecological zones along PC1 was primarily driven by reducing sugars, moisture content, and sucrose, whereas PC2 was mainly associated with diastase number, free acidity, and pH. Mountain honey samples were positioned in the direction of higher diastase activity and free acidity, while foothill and steppe samples were more strongly associated with higher reducing sugar content. Semi-desert samples were characterized by relatively higher pH values and lower moisture content, contributing to their separation from the remaining ecological zones.

**Figure 3:**
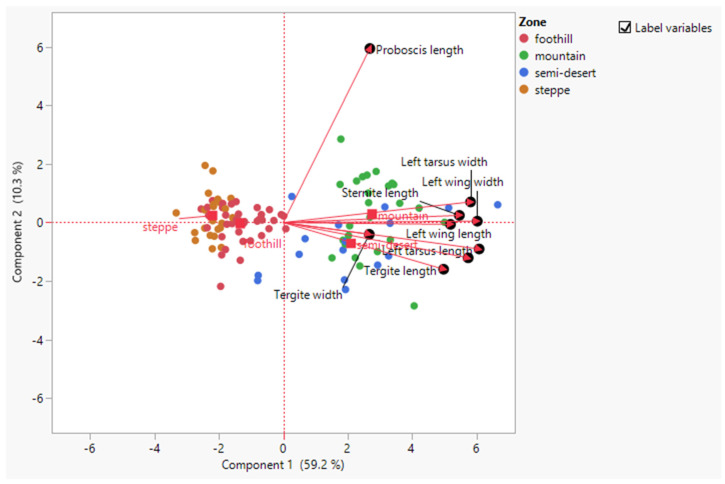
Principal component analysis (PCA) biplot of honey bee morphometric parameters across ecological zones.

Principal component analysis of the morphometric traits demonstrated that the first principal component (PC1) explained 59.2% of the total variance, whereas the second principal component (PC2) explained 10.3%, together accounting for 69.5% of the overall variability (Figure 3).

The loading matrix revealed that left tarsus width (0.325), left tarsus length (0.324), right tarsus width (0.315), left wing width (0.313), sternite width (0.293), wax mirror width (0.294), sternite length (0.291), wax mirror length (0.289), and right tarsus length (0.274) exhibited the highest positive loadings on PC1. This indicates that PC1 primarily represented overall body size and appendage dimensions. In contrast, PC2 was mainly influenced by hamuli count (0.544) and left tarsal index (0.491), whereas the right tarsal index (−0.427) contributed in the opposite direction. PC2 primarily reflected variation in wing venation and tarsal proportions rather than overall body size.

The loading plot demonstrated that proboscis length, forewing length, and cubital index contributed most strongly to PC1, whereas wing width and the hantel index showed the greatest contributions to PC2. Mountain populations were primarily associated with larger body size and longer proboscis length, while steppe and semi-desert populations exhibited comparatively smaller morphometric measurements. These variables therefore represented the principal drivers underlying morphometric differentiation among ecological zones.

Principal component analysis of the morphometric indices demonstrated a very high degree of data representation within the first two principal components. PC1 explained 68.61% of the total variance, whereas PC2 explained 26.41%, together accounting for 95.02% of the overall variability. This indicates that the two-dimensional PCA biplot captured nearly all of the variation present in the morphometric index dataset.

The loading matrix showed that cubital index (0.957) exhibited the strongest positive loading on PC1, followed by hantel index (0.784) and discoidal shift (0.726). Therefore, PC1 primarily represented the overall variation in wing venation characteristics. In contrast, PC2 was mainly associated with discoidal shift (0.667) in the positive direction and hantel index (−0.589) in the negative direction, indicating that these two indices were principally responsible for the secondary differentiation among ecological zones.

**Figure 4:**
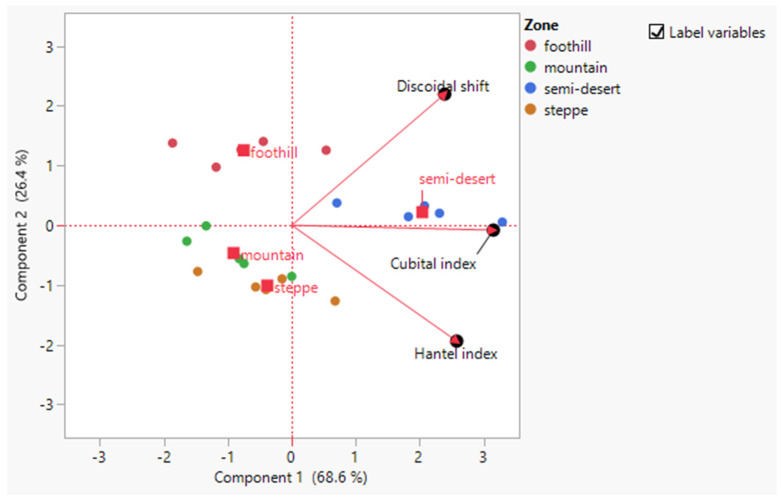
Principal component analysis (PCA) biplot of honey bee morphometric indices across ecological zones.

Projection of the ecological zones onto the PCA space showed that semi-desert populations were clearly separated along the positive direction of PC1, reflecting higher values of the morphometric indices, whereas mountain, foothill, and steppe populations were positioned on the negative side of PC1 and differed primarily along PC2 according to variation in the hantel index and discoidal shift. These results indicate that wing venation indices represented the principal morphometric characteristics contributing to ecological differentiation (Figure 4).

For the integrated PCA, the strongest positive loadings on PC1 were observed for diastase number, proboscis length, and free acidity, whereas reducing sugars and moisture contributed most strongly in the opposite direction. PC2 was largely influenced by pH and sucrose content. These loading patterns indicate that both honey physicochemical characteristics and selected morphometric traits jointly contributed to the differentiation of ecological zones, with enzymatic activity and morphometric adaptation representing the dominant sources of variation.

PCA of morphometric parameters explained 69.5% of the total variance (PC1 = 59.2%, PC2 = 10.3%). PC1 represented a general body size gradient, strongly influenced by wing length, wing width, tarsus length, and sternite and tergite dimensions. PC2 was mainly driven by proboscis length, reflecting functional adaptation related to nectar foraging.

Compared to physicochemical variables, separation among ecological zones was less pronounced. Mountain and semi-desert samples tended to exhibit larger body dimensions, whereas steppe samples clustered towards smaller sizes. Foothill samples again showed high variability and overlap with other zones.

A comparative analysis of the three PCA models revealed clear differences in their discriminatory power. The physicochemical dataset provided strong ecological differentiation, reflecting the dominant influence of environmental conditions on honey composition. Raw morphometric parameters showed moderate variation and limited separation, indicating relatively homogeneous body structure across zones.

**Figure 5:**
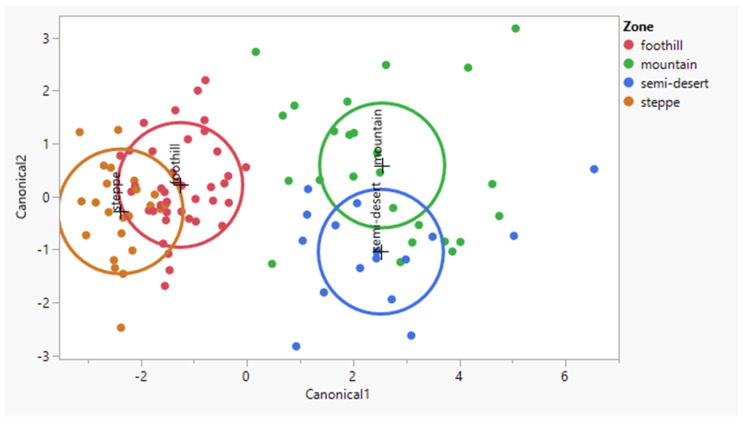
Linear discriminant analysis (LDA) canonical plot for classification of honey samples across ecological zones.

LDA was performed to evaluate the classification of honey samples according to ecological zones. The model demonstrated an overall classification accuracy of 74.76%, with a misclassification rate of 25.24%. The entropy R^2^ value (0.6598) indicates a satisfactory discriminatory performance of the model (Figure 5).

The canonical plot revealed clear separation among ecological zones, particularly between steppe and semi-desert samples, which formed distinct clusters. Mountain samples were moderately separated but showed partial overlap with semi-desert samples, while foothill samples exhibited the greatest dispersion and overlap with other zones.

Classification results showed that steppe samples were most accurately identified (82.6% correct classification), followed by foothill samples (79.5%). Semi-desert samples showed moderate classification accuracy (75.0%), whereas mountain samples exhibited lower accuracy (60.0%), reflecting overlap with adjacent ecological zones.

The leave-one-out cross-validation procedure demonstrated good predictive performance of the discriminant model, with an overall cross-validated classification accuracy of 74.76%. The corresponding confusion matrix is presented in Supplementary Table S2. Most samples were correctly assigned to their respective ecological zones, whereas the remaining misclassifications occurred primarily between ecologically adjacent zones, indicating gradual environmental transitions rather than sharply defined ecological boundaries.

## 4. Discussion

The present study should be interpreted within the context of ecological variation rather than direct cause-and-effect relationships between honey bee morphology and honey composition. Honey physicochemical properties are known to be influenced by multiple interacting factors, including botanical origin, climatic conditions, altitude, and beekeeping management. Honey bee morphometric traits were therefore evaluated as indicators of ecological adaptation that may be associated with regional variation in honey characteristics rather than as independent determinants of honey composition. The associations identified in the present study should not be interpreted as evidence that individual morphometric traits directly alter honey physicochemical composition. Rather, these traits likely reflect ecological adaptation of honey bee populations inhabiting different environmental conditions, where climatic factors, altitude, and regional vegetation collectively influence nectar resources and, consequently, honey characteristics.

The physicochemical characteristics of honey are fundamentally shaped by the environmental conditions prevailing in the region of production, including altitude, temperature regime, precipitation, and floral diversity [1,3]. The results of the present study demonstrate clear and statistically significant gradients in honey quality parameters across four ecological zones of Kazakhstan—mountain, foothill, steppe, and semi-desert—and are broadly consistent with patterns reported for other continental and highland honey-producing regions [14,26,27,28].

Moisture content, which ranged from 15.8 ± 1.2% in mountain honeys to 17.2 ± 2.0% in steppe honeys, remained well within the maximum permissible limit of 20% set by the Codex Alimentarius standard [22]. The significantly higher moisture in steppe samples can be attributed to the semi-continental climate of the Kazakh steppe, characterized by wider diurnal humidity fluctuations and generally higher ambient humidity during the nectar-ripening period compared to more arid or alpine environments [26]. A comparable moisture gradient related to altitude and climate zone has been reported for Algerian highland honeys, where samples from lower-elevation, hotter zones showed consistently elevated moisture relative to mountain counterparts [27]. Similarly, Habib et al. [28] found that honeys from arid regions of the United Arab Emirates had relatively lower moisture than those from more temperate origins, attributing this difference to accelerated nectar dehydration under dry, high-temperature conditions. The steppe zone of Kazakhstan, despite its continental aridity, experiences summer precipitation episodes and lower evaporation efficiency during honey ripening at the hive level, potentially accounting for the observed moisture elevation. All values recorded in the present study are compliant with international quality norms, indicating that honey from Kazakh ecological zones represents a product of acceptable moisture status irrespective of origin.

Reducing sugar content showed the most pronounced inter-zone differences, ranging from 72.5 ± 5.8% in semi-desert honeys to 82.7 ± 8.9% in foothill samples. The high reducing sugar content in foothill honeys likely reflects the rich diversity of glucose- and fructose-rich floral sources characteristic of this transitional landscape, including fruit orchards, meadow flora, and early-blooming Rosaceae species whose nectars are known to be sugar-rich [3,29]. In contrast, the lower reducing sugar content in semi-desert honeys may be associated with the prevalence of xerophytic flora, including *Tamarix* spp. and *Artemisia* spp., which produce nectars with modified sugar profiles compared to mesophytic species [13]. Ongalbek et al. [13], studying buckwheat and monofloral Kazakh honeys by chemometric classification, similarly reported significant variation in sugar profiles among honey types from different ecological contexts in Kazakhstan, with botanical origin exerting a strong effect on fructose-to-glucose ratios and total reducing sugar content. The observation that sucrose content did not differ significantly among zones (range 2.4–3.8%, *p* > 0.05) and remained well below the Codex 5% threshold [22] indicates complete or near-complete enzymatic hydrolysis of sucrose in all samples, irrespective of ecological origin, and confirms product maturity across the dataset.

Free acidity was the highest in mountain honeys (30.5 ± 5.2 meq/kg) and decreased progressively toward the semi-desert zone (22.1 ± 6.3 meq/kg), while pH followed the inverse trend (4.1 in mountain vs. 4.5 in semi-desert). These gradients are consistent with the well-established relationship between botanical origin and organic acid composition in honey. Mountain floral communities dominated by *Apiaceae, Lamiaceae*, and *Rosaceae* species produce nectars rich in malic, citric, and gluconic acid precursors, which contribute to elevated free acidity and lower pH in the resulting honey [1,4]. Bouhala et al. [27] reported a similar altitude-driven acidity gradient for Algerian honeys, with mountain samples showing significantly higher titratable acidity than low-elevation equivalents, and attributed this pattern to the prevalence of polleniferous mountain flora producing organically richer nectars. Suleiman et al. [26], analyzing honeys from different altitudinal zones in the Arabian Peninsula, likewise documented lower pH and higher phenolic-linked acidity in highland honeys compared to lowland samples. The free acidity values recorded here for Kazakh mountain honeys (30.5 meq/kg) are within the internationally permitted maximum of 50 meq/kg [22] and are consistent with the typical range for high-quality European and Central Asian monofloral honeys [1,3].

Diastase activity showed the most ecologically informative gradient among all physicochemical parameters, declining from 18.4 ± 6.3 Schade units in mountain honeys to 10.8 ± 4.2 in semi-desert samples (*p* < 0.05). All values exceeded the minimum threshold of 8 DN required by the Codex Alimentarius for honey not subjected to excessive heating [22]. This gradient can be interpreted through two complementary mechanisms. First, lower ambient temperatures in mountain environments slow the post-harvest degradation of diastase, a heat-labile enzyme, resulting in higher residual activity at the point of sampling [2,30]. Pasias et al. [2], in a comprehensive study of diastase activity in Greek honeys, demonstrated that diastase is highly sensitive to both storage temperature and initial enzymatic load, with both factors operating simultaneously in field samples. In the present study, honey from mountain apiaries was harvested and stored under cooler conditions (mean daily temperature during August sampling ca. 12–18 degrees C at elevations above 1500 m), which would retard diastase denaturation relative to steppe and semi-desert conditions (ca. 28–34 degrees C). Second, the botanical composition of mountain flora, particularly the prevalence of high-nectar-secreting Phacelia, Echium, and Astragalus species, is associated with inherently higher salivary gland enzyme secretion by forager bees, potentially resulting in a higher initial diastase inoculation into nectar [30,31]. Nayik et al. [31], studying high-altitude Indian honeys, found that diastase activity was consistently higher in samples from elevations above 1500 m, regardless of botanical origin, and associated this with both nectar enrichment and reduced thermal degradation—a mechanistic framework directly applicable to the Kazakh montane context. Moldakhmetovaet al. [14] likewise recorded higher enzymatic activity in honey from mountain climate zones of Kazakhstan compared to steppe and desert origins, providing direct regional corroboration of the gradient observed here.

The morphometric analysis revealed significant inter-zone variation in most body measurements of *Apis mellifera* workers, with mountain and semi-desert bees consistently exhibiting larger body dimensions than foothill and steppe counterparts. Proboscis length was greatest in mountain bees (6.69 ± 0.58 mm), followed by a markedly smaller and statistically indistinguishable cluster in foothill (6.23 ± 0.29 mm), steppe (6.24 ± 0.35 mm), and semi-desert (6.21 ± 0.59 mm) zones. Wing length, sternite dimensions, and tarsus measurements showed a broadly bimodal pattern, with mountain and semi-desert populations larger than foothill and steppe, while the tarsal index and hamuli count showed no significant zone differentiation.

The pattern of larger body size in mountain bees is consistent with predictions derived from Bergmann’s rule, which postulates that endothermic and ectothermic organisms at higher latitudes or altitudes tend toward larger body size as an adaptation to thermal conservation [32,33]. In bees specifically, Gerard et al. [32] demonstrated, across a continental dataset spanning Europe and North Africa, that body size increases with latitude in a pattern broadly consistent with Bergmann’s rule, with cold-adapted populations exhibiting significantly larger wing surfaces and body volumes. Osorio-Canadas et al. [33], analyzing temporal variation in bee body size in a Mediterranean regional fauna, similarly found that larger individuals predominate in cooler periods and cooler microclimates, providing mechanistic evidence that thermoregulatory selection drives body size clines in bees. The mountain bees of Kazakhstan, which contend with cool temperatures, short foraging seasons, and energetically demanding high-altitude flight, may therefore represent a locally adapted ecotype in which large body size confers thermoregulatory and flight-performance advantages [33].

The comparably large body dimensions of semi-desert bees present a more nuanced ecological interpretation. Semi-desert environments impose severe physiological constraints on bees, including extreme heat, low floral density, and long foraging distances to reach water and sparse nectar sources [22]. Under these conditions, larger body size, particularly larger wing area and tarsus dimensions, may confer advantages in long-distance flight efficiency and water retention. Cariveau et al. [34], in their analysis of the allometry of bee proboscis length and body size across ecological gradients, noted that hot, low-resource environments can also select for larger body size when foraging range is a premium, albeit through different selective pressures than cold-environment selection. Gerard et al. [32], in a parallel study examining wing shape and health in stressed bumblebee populations, found that environmentally stressed bees show altered wing dimensions that are ecologically interpretable at the population level, suggesting that morphometric divergence reflects ecological adaptation rather than random variation. The bimodal pattern observed here—mountain and semi-desert bees larger than steppe and foothill bees—thus appears to reflect two contrasting but convergent adaptive responses to environmental extremes operating at opposite ends of the thermal and resource-availability spectrum.

The steppe and foothill zones, which support more moderate thermal conditions and relatively rich floral resources, showed smaller body dimensions consistent with less intense thermoregulatory or foraging-range selection. Foothill bees showed intermediate values for most parameters, reflecting the transitional ecological character of this zone. The non-significant variation in hamuli count and tarsal index across zones is noteworthy: both traits show limited plasticity and may be more strongly constrained by genetic factors than by phenotypic plasticity in response to environmental conditions [35]. Scriven et al. [35], studying body size variation in cryptic bumblebee species, found that certain morphometric traits are highly heritable and under strong stabilizing selection, while others are more responsive to environmental variation, a distinction that likely applies to *Apis mellifera* as well.

Regarding subspecies identity, Kazakhstan harbors a historically complex bee population structure. Sheppard and Meixner [15] described *Apis mellifera pomonella* as a distinct subspecies from the Tien Shan mountain range of Central Asia, distinguished from Western lineage subspecies by a suite of morphometric characters including the cubital index, discoidal shift, and body measurements. The most recent comprehensive morphometric and molecular survey of Kazakh honey bee populations confirmed the persistence of morphological A. m. pomonella-type individuals in mountain and foothill zones of south-eastern Kazakhstan while reporting mixed or introgressed population structures in steppe and semi-desert zones that reflect historical hybridization with introduced A. m. carnica stock. The larger proboscis and body dimensions of mountain bees in the present study are broadly consistent with the morphometric profile of A. m. pomonella as characterized by Sheppard and Meixner [15], while the steppe populations with their smaller dimensions may reflect a higher proportion of A. m. carnica ancestry. Sheralieva et al. [18] explicitly documented variation in the cubital index, hantel index, and discoidal shift among *Apis mellifera carnica* colonies maintained across Kazakh climate zones, finding that morphometric parameters varied systematically with ecological zone, further supporting the idea that the patterns in the present study reflect both phenotypic plasticity and genetic population structure. Chen et al. [36], in a broader genomic study of temperate honey bee adaptation across Central Asia, demonstrated that populations from montane Central Asian environments carry genomic signatures of adaptation to cold, short-season conditions distinct from those of steppe-dwelling populations, providing a molecular underpinning for the morphometric differences observed here.

The three derived morphometric indices—cubital index (CI), hantel index, and discoidal shift—showed distinct patterns of inter-zone variation that complement the raw measurement data. The cubital index increased progressively from mountain (2.66 ± 0.04) to semi-desert (2.90 ± 0.06) zones (*p* < 0.05), tracing a clear ecological gradient. The CI measures the ratio of anterior wing vein segments a and b and is one of the most taxonomically informative morphometric indices for *Apis mellifera* subspecies discrimination [10,16]. Values below 2.5 are typically associated with African and south-eastern European subspecies, while values in the 2.5–3.0 range are characteristic of the Western European and Central Asian lineages, with higher values generally found in populations from drier, hotter environments [10,37].

The significant associations observed between ecological zones, morphometric characteristics, and honey physicochemical properties should not be interpreted as evidence that morphometric traits directly determine honey composition. Instead, both groups of variables likely respond to the same underlying environmental gradients. For example, altitude, temperature, precipitation, and regional floral resources influence nectar availability, nectar composition, and honey maturation while simultaneously shaping the long-term ecological adaptation of local honey bee populations, which is reflected in morphometric characteristics such as the cubital index and sternite dimensions. Consequently, the observed clinal variation in the cubital index from mountain to semi-desert regions should be interpreted as an indicator of population adaptation to contrasting environmental conditions rather than a direct mechanistic driver of variation in honey moisture or reducing sugar content. The statistical associations identified in this study therefore most likely reflect shared responses to ecological gradients rather than direct cause-and-effect relationships.

Several limitations of the present study should be acknowledged. One limitation of this study is the absence of melissopalynological analysis to determine the botanical origin of individual honey samples. Consequently, the relationships identified in the present work should be interpreted at the ecological zone level rather than at the level of specific floral sources. Future studies integrating pollen analysis together with physicochemical, morphometric, and molecular data will provide a more comprehensive understanding of regional honey variability. An additional limitation of this study relates to the assumptions underlying MANOVA. Diagnostic testing indicated departures from multivariate normality and homogeneity of covariance matrices among ecological zones. Although MANOVA is considered relatively robust in balanced experimental designs, particularly when supported by complementary multivariate methods, these results should be interpreted as exploratory. Future studies with larger sample sizes and alternative robust multivariate approaches will further strengthen the statistical inference.

## 5. Conclusions

The present study demonstrates that ecological zones are associated with significant variation in honey physicochemical characteristics. Honey bee morphometric parameters exhibited comparatively limited variability but contributed to multivariate differentiation when analyzed together with ecological variables. These findings improve our understanding of regional honey variability in Kazakhstan while highlighting the importance of incorporating botanical origin through melissopalynological analysis in future studies. Honey bee morphometric traits—particularly proboscis length, sternite length, and cubital index—act as secondary modulators of honey composition in a zone-specific context, as evidenced by significant MANOVA interaction terms. The observed morphometric clines, including the progressive increase in the cubital index from mountain (2.66) to semi-desert (2.90) zones and the bimodal body-size pattern (larger mountain and semi-desert bees flanking smaller steppe and foothill bees), are consistent with ecogeographic adaptation consistent with Bergmann’s rule and with the historical population structure of Kazakh honey bees, which includes an endemic A. m. pomonella core in mountain zones partially overlaid by A. m. carnica introgression in lowland zones. The LDA achieved 74.76% cross-validated zone classification accuracy using combined physicochemical and morphometric variables, establishing a scientific basis for honey geographical origin authentication in Kazakhstan and identifying diastase activity, free acidity, and reducing sugar content as the most discriminatory parameters. These findings contribute to the growing body of evidence that honey quality in climatically diverse regions reflects the integrated outcome of environmental conditions, botanical composition, and the biological characteristics of the bee population and provide a quantitative foundation for evidence-based honey authentication, beekeeping management, and conservation of native bee populations in Kazakhstan.

## Figures and Tables

**Table 1:** Physicochemical properties of honey across ecological zones.

Zone	Moisture (%)	Reducing Sugars (%)	Sucrose (%)	Free Acidity (meq/kg)	pH	Diastase (DN)
Mountain	15.8 ± 1.2 b	80.2 ± 4.5 b	2.4 ± 0.6 a	30.5 ± 5.2 b	4.1 ± 0.2 b	18.4 ± 6.3 a
Foothill	16.3 ± 1.5 b	82.7 ± 8.9 a	3.1 ± 1.2 a	28.7 ± 7.1 b	4.2 ± 0.3 b	16.5 ± 7.8 a
Steppe	17.2 ± 2.0 a	75.6 ± 6.2 c	3.8 ± 1.5 a	24.3 ± 8.9 c	4.3 ± 0.4 a	11.2 ± 5.4 b
Semi-desert	16.0 ± 1.4 b	72.5 ± 5.8 c	2.9 ± 0.9 a	22.1 ± 6.3 c	4.5 ± 0.3 a	10.8 ± 4.2 b

Values are presented as mean ± standard deviation (SD). Different letters within the same row indicate significant differences among ecological zones according to Tukey’s honestly significant difference (HSD) test following one-way ANOVA (*p* < 0.05). DN, diastase number.

**Table 2:** Morphometric characteristics of worker honey bees from different ecological zones of Kazakhstan.

Zone	Mountain	Foothill	Steppe	Semi-Desert
Proboscis length (mm)	6.69 ± 0.58 a	6.23 ± 0.29 b	6.24 ± 0.35 b	6.21 ± 0.59 b
Hamuli count	21.04 ± 1.34 a	21.28 ± 1.23 a	21.13 ± 1.29 a	20.88 ± 1.46 a
Tergite length (mm)	2.49 ± 0.25 a	2.18 ± 0.11 b	2.05 ± 0.09 b	2.40 ± 0.26 ab
Tergite width (mm)	4.99 ± 0.52 a	4.84 ± 0.28 a	4.81 ± 0.14 a	4.98 ± 0.30 a
Sternite length (mm)	3.18 ± 0.32 a	2.74 ± 0.07 b	2.72 ± 0.08 b	3.21 ± 0.30 a
Sternite width (mm)	4.77 ± 0.39 a	4.29 ± 0.20 b	3.98 ± 0.12 c	4.86 ± 0.31 a
Wax mirror length (mm)	1.73 ± 0.15 a	1.49 ± 0.06 b	1.48 ± 0.07 b	1.64 ± 0.15 a
Wax mirror width (mm)	2.77 ± 0.22 a	2.34 ± 0.13 b	2.31 ± 0.10 b	2.71 ± 0.22 a
Tarsus length (mm)	2.57 ± 0.12 a	2.30 ± 0.22 ab	2.09 ± 0.06 b	2.51 ± 0.15 a
Tarsus width (mm)	1.44 ± 0.03 a	1.23 ± 0.08 b	1.18 ± 0.04 b	1.35 ± 0.09 a
Tarsal index	56.37 ± 2.97 a	54.13 ± 6.78 a	56.40 ± 2.16 a	56.13 ± 1.98 a
Wing length (mm)	9.77 ± 0.27 a	9.39 ± 0.22 b	9.34 ± 0.17 b	9.71 ± 0.32 a
Wing width (mm)	3.79 ± 0.26 a	3.11 ± 0.15 b	3.10 ± 0.12 b	3.72 ± 0.25 a

Data are expressed as mean ± standard deviation (SD). Means within the same row followed by different letters (a–c) are significantly different according to Tukey’s honestly significant difference (HSD) post hoc test after one-way ANOVA (*p* < 0.05). All morphometric measurements are expressed in millimeters (mm), except hamuli count (number of wing hooks) and the tarsal index (dimensionless).

**Table 3:** Morphometric indices of worker honey bees from different ecological zones of Kazakhstan.

Zone	Cubital Index (CI)	Hantel Index	Discoidal Shift (DS)
Mountain	2.66 ± 0.04 c	1.08 ± 0.01 a	3.84 ± 0.16 c
Foothill	2.71 ± 0.07 b	1.06 ± 0.01 a	4.06 ± 0.29 b
Steppe	2.78 ± 0.05 b	1.08 ± 0.01 a	3.71 ± 0.14 c
Semi-desert	2.90 ± 0.06 a	1.09 ± 0.01 a	4.23 ± 0.18 a

Values are presented as mean ± standard deviation (SD). Different letters within the same row indicate significant differences among ecological zones according to Tukey’s honestly significant difference (HSD) test following one-way ANOVA (*p* < 0.05). CI, cubital index; DS, discoidal shift. The Hantel index is dimensionless.

**Table 4:** Multivariate effects of ecological zone, morphometric traits, and morphometric indices on honey physicochemical properties (MANOVA, Wilks’ Lambda).

Effect	Wilks’ Λ	F	df (num, den)	*p*-Value
Whole model (traits + indices)	0.16	1.11	144, 434.5	0.214
Ecological zone	0.853	0.66	18, 206.9	0.844
Zone × Proboscis length	0.782	1.05	18, 206.9	0.0411
Zone × Tergite length	0.809	0.89	18, 206.9	0.587
Zone × Tergite width	0.869	0.59	18, 206.9	0.907
Zone × Sternite length	0.657	1.84	18, 206.9	0.0227
Zone × Sternite width	0.832	0.77	18, 206.9	0.731
Zone × Left wing length	0.841	0.73	18, 206.9	0.78
Zone × Left wing width	0.868	0.59	18, 206.9	0.905
Zone × Cubital index	0.008	1.51	18, 6.14	0.0317
Zone × Hantel index	0.029	0.86	18, 6.14	0.634
Zone × Discoidal shift	0.015	1.16	18, 6.14	0.459

## Data Availability

The original contributions presented in this study are included in the article/Supplementary Materials. Further inquiries can be directed to the corresponding author.

## Supplementary Materials

The following supporting information can be downloaded at: https://www.mdpi.com/article/10.3390/foods15142454/s1, Table S1: Geographic location, ecological zone, altitude above sea level, and sampling information for the 103 honey samples collected from apiaries across Kazakhstan; Table S2: Leave-one-out cross-validation (LOOCV) confusion matrix and classification accuracy of the linear discriminant analysis (LDA) for ecological zone classification of the 103 honey samples.

## Abbreviations

The following abbreviations are used in this manuscript:

ANOVA	Analysis of Variance
CI	Cubital Index
DN	Diastase Number
DS	Discoidal Shift
IHC	International Honey Commission
LDA	Linear Discriminant Analysis
MANOVA	Multivariate Analysis of Variance
PCA	Principal Component Analysis
