# Supplementary material for: Influence of Ecological Zones and Honey Bee Morphometric Traits on the Physicochemical Properties of Honey in Kazakhstan
Source: Foods. 2026 Jul 10;15(14):2454. doi: 10.3390/foods15142454 (PMC13408582; doi:10.3390/foods15142454)
Supplement: Supplementary file 1 [file foods-15-02454-s001.zip › foods-4380746-supplementary.pdf]

Table S1. Geographic location, ecological zone, altitude above sea level, and sampling information for the 103 honey samples collected from apiaries across Kazakhstan.

Sample No.	Region	District/Location	Farm/Apiary	Climatic Zone	Latitude	Longitude	Sea level (m a.s.l.)
1	West Kazakhstan Region	Baiterek district	Batayeva	Steppe	51.28907174179334	50.93720079603993	62
2	West Kazakhstan Region	Baiterek district	Batayeva	Steppe	51.28907174179334	50.93720079603993	70
3	West Kazakhstan Region	Baiterek district	Batayeva	Steppe	51.28907174179334	50.93720079603993	77
4	Almaty Region	Enbershikazakh district, Malovodnoe village		Foothill	43.51430561839595	77.68910689545653	715
5	Abai Region	Urzhar district	Vladik	Steppe	47.09681529255643	81.62937436807081	465
6	Abai Region	Urzhar district	Vladik	Steppe	47.09681529255643	81.62937436807081	535
7	Abai Region	Urzhar district	Madi	Steppe	47.09681529255643	81.62937436807081	590
8	Zhetysu Region	Kerbulak district	Panav	Foothill	44.50986229849464	78.89729160712815	720
9	Zhetysu Region	Kerbulak district	Panav	Foothill	44.50986229849464	78.89729160712815	825
10	Abai Region	Urzhar district, Karatuma village	Krason	Foothill	47.08700880191086	81.63148018208936	715
11	Abai Region	Urzhar district, Karatuma village	Krason	Foothill	47.087081853103676	81.63155528393624	795
12	Almaty Region	Alma-Arasan Gorge	Almarasan	Mountain	43.09057002968076	76.90601365231748	1760
13	Almaty Region	Alma-Arasan Gorge	Almarasan	Mountain	43.09057002968076	76.90601365231748	1803
14	Almaty Region	Zhambyl district, Kastek village	Kastek	Foothill	43.133814599926126	75.84197105164712	1210
15	Almaty Region	Zhambyl district, Kastek village	Kastek	Foothill	43.133814599926126	75.84197105164712	1315
16	Almaty Region	Zhambyl district, Kastek village	Kastek	Foothill	43.133814599926126	75.84197105164712	1375

17	Almaty Region	Enbekshikazakh district, Bolek village	Mahymet	Steppe	43.407853011788006	77.4215554769672 4	640
18	Almaty Region	Enbekshikazakh district, Bolek village	Mahymet	Steppe	43.407853011788006	77.4215554769672 4	695
19	Almaty Region	Karasai district, Kyrgauyldy village	Rudenko Evgeniy	Foothill	43.180249711811214	76.7667873768526	850
20	Almaty Region	Karasai district, Kyrgauyldy village	Ruslan	Foothill	43.180249711811214	76.7667873768526	905
21	Kostanai Region	Saranov district, Lisakovsk town	Jusip	Steppe	52.5462491626418	62.495725259712614	195
22	North Kazakhstan Region	Petropavl city	Stepura	Steppe	54.89558876369372	69.12165079398437	128
23	North Kazakhstan Region	Petropavl city	Stepura	Steppe	54.89558876369372	69.12165079398437	147
24	Zhetysu Region	Eskeldy district, Tekely town	Kudryashev	Mountain	44.870340180731354	78.78463593812732	1191
25	Zhetysu Region	Eskeldy district, Tekely town	Panav	Mountain	44.870340180731354	78.78463593812732	1241
26	Zhetysu Region	Eskeldy district, Tekely town	Kozlov	Mountain	44.8703401807313	78.78463593812732	895
27	Zhetysu Region	Eskeldy district, Tekely town	Zhambotau honey farm	Foothill	44.7969221380887	78.8946650735036	970
28	Zhambyl Region	Merke district, Merke town	Pastukh farm	Foothill	42.8713386064955	73.1965730782781	875
29	Zhetysu Region	Eskeldy district, Tekely town	Mykhmet farm	Mountain	44.8435317667316	78.8234988519077	1286
30	Zhetysu Region	Eskeldy district, Tekely town	Mykhmet farm	Mountain	44.8435317667316	78.8234988519077	1341
31	Turkistan Region	Kazygurt district, Kazygurt village	Shatskikh farm	Foothill	41.7544557319605	69.3847983099514	860
32	Zhetysu Region	Eskeldy district, Tekely town	Rudenko Evgeniy	Foothill	44.8435317667316	78.8234988519077	1020
33	Zhetysu Region	Eskeldy district, Tekely town	Panov	Mountain	44.870340180731354	78.78463593812732	1396
34	Zhetysu Region	Eskeldy district, Tekely town	Panov	Mountain	44.870340180731354	78.78463593812732	1451
35	Zhetysu Region	Eskeldy district, Tekely town	Kudreshov	Foothill	44.870340180731354	78.78463593812732	1065
36	Zhetysu Region	Eskeldy district, Tekely town	Kudreshov	Foothill	44.870340180731354	78.78463593812732	1100

37	Almaty Region	Enbekshikazakh district, Saimasai village	Saimasai farm	Foothill	43.4416859049549	77.3359030581667	740
38	Turkistan Region	Kazygurt district, Zhegirgen village	Darkhan farm	Semi-desert	41.908822862654	69.7327994388476	935
39	Turkistan Region	Maktaaral district, Zhetisay	Golovashkin farm	Semi-desert	40.775262978372766	68.33811848371383	227
40	Turkistan Region	Maktaaral district, Zhetisay	Golovashkin farm	Semi-desert	40.775262978372766	68.33811848371383	240
41	Turkistan Region	Maktaaral district, Zhetisay	Aisultan farm	Semi-desert	40.775262978372766	68.33811848371383	250
42	Turkistan Region	Maktaaral district, Zhetisay	Yntymak bal	Semi-desert	40.775262978372766	68.33811848371383	263
43	Turkistan Region	Maktaaral district, Zhetisay	Duisebay	Semi-desert	40.775262978372766	68.33811848371383	273
44	Turkistan Region	Maktaaral district, Zhetisay	Melekhov	Semi-desert	40.7752629783727	68.3381184837138	285
45	Karaganda Region	Karaganda city	Balkhash farm	Steppe	49.7604776447865	73.1243279633283	545
46	Almaty Region	Uygur district, Chundzha town	Marup farm	Foothill	43.3749813780157	79.5923420091413	665
47	Zhambyl Region	Kordai district, Karakonyz Gorge	Manatbek #1	Mountain	42.9749485994196	75.5645486119042	1330
48	Zhambyl Region	Kordai district, Karakonyz Gorge	Manatbek #1	Mountain	42.97494859941965	75.56454861190429	1415
49	Almaty Region	Almaty city, Alma-Arasan Gorge	Alma-Arasan honey farm	Mountain	43.089686386525834	76.91109084301598	1827
50	Kostanai Region	Rudnyi city	Petrovskaya farm	Steppe	52.972105985727644	63.13998326350794	230
51	Turkestan Region	Kazygurt district, Zhegirgen village	Darkhan farm	Semi-desert	41.9093658058673	69.7332285922585	1015
52	East Kazakhstan Region	Oskemen city	Vshoz LLP	Mountain	49.953596636249195	82.66395512785412	1025
53	Turkistan Region	Saryagash disctrict, Saryagash town	Alyaev P. farm	Semi-desert	41.464300828892775	69.2106484151379	425
54	Almaty Region	Eskeldy district, Tekely town	Mahmet	Mountain	44.8435317667316	78.8234988519077	1496
55	Turkistan Region	Tulkibas district	Koroleva sot farm	Semi-desert	42.48524583261177	70.40653288368358	600
56	Abai Region	Semei city	Nash med 2 farm	Steppe	50.4195199596971	80.3060382352302	200
57	Almaty Region	Enbekshikazakh district, Esik town	Zhuravleva farm	Foothill	43.3560296943135	77.4614184361404	960
58	Zhambyl Region	Merke district, Merke town	Galyuk	Semi-desert	42.8713386064955	73.1965730782781	650
59	Zhambyl Region	Merke district, Merke town	Vladimir zhantak	Semi-desert	42.8713386064955	73.1965730782781	700

60	East Kazakhstan Region	Shemonaiha district, Pervomaiskaya village	Emashov farm	Mountain	50.25973943742025	81.99371323985122	1005
61	East Kazakhstan Region	Shemonaiha district, Pervomaiskaya village	Emashov farm	Mountain	50.25973943742025	81.99371323985122	1048
62	East Kazakhstan Region	Shemonaiha district, Pervomaiskaya village	Emashov farm	Mountain	50.25973943742025	81.99371323985122	1082
63	East Kazakhstan Region	Shemonaiha district, Pervomaiskaya village	Emashov farm	Mountain	50.25973943742025	81.99371323985122	1115
64	Zhambyl Region	Kordai district, Karakonyz Gorge	Baizhigitov farm	Mountain	42.97494859941965	75.56454861190429	1530
65	Zhambyl Region	Merke district, Merke town	Saranchuk Vladimir	Semi-desert	42.8713386064955	73.1965730782781	755
66	Turkestan Region	Maktaaral district, Zhetisay	Golovashkin hlopok	Semi-desert	40.780675022916896	68.3378838596166	297
67	Turkestan Region	Maktaaral district, Zhetisay	Golovashkin	Semi-desert	40.780675022916896	68.3378838596166	310
68	Turkestan Region	Maktaaral district, Zhetisay	Sambetov	Foothill	40.780675022916896	68.3378838596166	685
69	Abai Region	Semei city	Nash med 1 farm	Steppe	50.423263622944226	80.25036797902135	235
70	North Kazakhstan Region	Petropavl city	Bogomolov	Steppe	54.884693776053	69.305620673786	158
71	East Kazakhstan Region	Glubokoe district	Paseka Bunkovykh	Steppe	50.294136993790026	82.85288633049056	430
72	Zhetysu Region	Eskeldy district, Tekely town	Kozlov farm	Mountain	44.86401070956334	78.98891961630058	1266
73	Almaty Region	Karasai district, Shamalgan village	Sugakov	Foothill	43.377186858102064	76.63278480125817	965
74	Turkistan Region	Maktaaral district, Zhetisay	Sattarov	Semi-desert	40.7806750229168	68.3378838596166	320
75	Almaty Region	Karasai district, Shamalgan village	Abay farm	Foothill	43.38695922465495	76.63418765751722	1015
76	Zhetysu Region	Alakol district, Tok-Zhailau village	Tleukhan	Mountain	43.412389	77.834444	1290
77	Turkestan Region	Tulkibas district, Shakpak baba village	Shakpak baba farm	Foothill	42.50975210565506	70.5592201007441	835
78	Turkistan Region	Saryagash disctrict, Saryagash town	Berik	Steppe	41.4634003593388	69.182925104798	480
79	Turkistan Region	Saryagash disctrict, Saryagash town	Alfiya farm	Steppe	41.4634003593388	69.182925104798	515

80	Zhetysu Region	Eskeldy district, Tekely town	Zhambotau honey farm	Foothill	44.7969221380887	78.8946650735036 2	895
81	Zhetysu Region	Alakol district, Ush bulak village	Fedosov farm	Mountain	45.8000621434321	81.3061867746524	1380
82	Zhetysu Region	Eskeldy district, Tekely town	Panov	Mountain	44.8703401807313	78.7846359381273	1371
83	Zhetysu Region	Alakol district, Tok-Zhailau village	Tleukhan	Mountain	43°24'44.6"N	77°50'04.0"E	1485
84	Zhetysu Region	Kerbulak district	Amir farm	Mountain	44.4172324442907	78.5486387160502	1100
85	Zhetysu Region	Alakol district, Tok zhailau	Koishibekov farm	Mountain	43°24'44.6"N	77°50'04.0"E	1570
86	Almaty Region	Uygur district, Chundzha	Marup	Foothill	43.3749813780157 4	79.5923420091413 2	745
87	Turkestan Region	Kazygurt district, Kazygurt village	Med Ainabekovykh	Foothill	41.760090139772	69.3872015690522	1075
88	Almaty Region	Zhambyl district, Akkainar village	Pchelka farm	Foothill	43.1715893899573	76.0441096963539	1065
89	Almaty Region	Uygur district	Shukin	Foothill	43.5311897641565 44	79.4453661850090 2	795
90	Almaty Region	Enbekshikazakh district, Kazakhstan village	Beequeen farm	Foothill	43.5246197205505 54	77.7806025683428	815
91	Kyzylorda Region	Syrdaria district	Glotova	Steppe	44.8873623834291	65.4022557616195	125
92	Almaty Region	Uygur district	Marup	Foothill	43.3749813780157 4	79.5923420091413 2	840
93	East Kazakhstan Region	Ulan district	Mayer	Steppe	49.7409969491071	82.2085965816749	440
94	Turkestan Region	Turkestan city	Shatskikh	Steppe	41.7544557319605	69.3847983099514	250
95	Zhetysu Region	Alakol district, Lepsi village	Lepsinsk onimi	Mountain	45.5437310298676	80.6659150683834	1630
96	Almaty Region	Zhambyl district	Pchelka raznotrav	Foothill	43.1718564717461	76.0435649478894	850
97	Kyzylorda Region	Syrdaria district	Glotova zhyngyl	Steppe	44.8873623834291	65.4022557616195	150
98	Almaty Region	Enbekshikazakh district, Malovodnoe	Zholshiev	Foothill	43.5143056183959 5	77.6891068954565 3	785
99	East Kazakhstan Region	Shemonaiha district, Pervomaiskaya village	Emashov gornoe	Mountain	50.2597394374202 5	81.9937132398512 2	1145
100	East Kazakhstan Region	Shemonaiha district, Pervomaiskaya village	Emashov akaci	Mountain	50.2597394374202 5	81.9937132398512 2	1175
101	Zhetysu Region	Alakol district, Zhailau village	Tleuhan gornoe	Mountain	43°24'44.6"N	77°50'04.0"E	1700
102	Zhetysu Region	Eskeldy district, Tekely town	Zhambotau	Mountain	44.7969221380887	78.8946650735036	1426
103	Zhetysu Region	Eskeldy district	Akkaiyn	Mountain	44.8073970522578	78.8678859006675	1170

Table S2. Leave-one-out cross-validation (LOOCV) confusion matrix and classification accuracy of the linear discriminant analysis (LDA) for ecological zone classification of the 103 honey samples.

**Actual \ Predicted**	**Foothill**	**Mountain**	**Semi-desert**	**Steppe**
**Foothill (39)**	**29**	3	5	2
**Mountain (25)**	2	**20**	2	1
**Semi-desert (16)**	2	1	**12**	1
**Steppe (23)**	3	2	2	**16**
